# Endometrial thickness on the day of the LH surge: an effective predictor of pregnancy outcomes after modified natural cycle-frozen blastocyst transfer

**DOI:** 10.1093/hropen/hoaa060

**Published:** 2020-12-17

**Authors:** Sachie Onogi, Kenji Ezoe, Seiko Nishihara, Junichiro Fukuda, Tamotsu Kobayashi, Keiichi Kato

**Affiliations:** Kato Ladies Clinic, Tokyo 160-0023, Japan

**Keywords:** endometrial thickness, frozen blastocyst transfer, LH surge, natural cycle, proliferation phase, live birth, GnRH agonist trigger, female aging

## Abstract

**STUDY QUESTION:**

Can the endometrial thickness (EMT) on the day of the LH surge predict pregnancy outcomes after single vitrified-warmed blastocyst transfers (SVBTs) in modified natural cycles?

**SUMMARY ANSWER:**

Decreased EMT on the day of the LH surge is associated with older female age and a shortened proliferation phase and may be associated with low live birth and high chemical pregnancy rates.

**WHAT IS KNOWN ALREADY:**

The relation between EMT on the day of embryo transfer (ET) and pregnancy outcomes remains controversial; although numerous studies reported an association between decreased EMT on the day of ET and a reduced likelihood of pregnancy, recent studies demonstrated that the EMT on the day of ET had limited independent prognostic value for pregnancy outcomes after IVF. The relation between EMT on the day of the LH surge and pregnancy outcomes after SVBT in modified natural cycles is currently unknown.

**STUDY DESIGN, SIZE, DURATION:**

In total, 808 SVBTs in modified natural cycles, performed from November 2018 to October 2019, were analysed in this retrospective cohort study. Associations of EMT on the days of the LH surge with SVBT and clinical and ongoing pregnancy rates were statistically evaluated. Clinical and ongoing pregnancy rates were defined as the ultrasonographic observation of a gestational sac 3 weeks after SVBTs and the observation of a foetal heartbeat 5 weeks after SVBTs, respectively. Similarly, factors potentially associated with the EMT on day of the LH surge, such as patient and cycle characteristics, were investigated.

**PARTICIPANTS/MATERIALS, SETTING, METHODS:**

The study includes IVF/ICSI patients aged 24–47 years, who underwent their first SVBT in the study period. After monitoring follicular development and serum hormone levels, ovulation was triggered via a nasal spray containing a GnRH agonist. After ovulation was confirmed, SVBTs were performed on Day 5. The EMT was evaluated by transvaginal ultrasonography on the day of the LH surge and immediately before the SVBT procedure.

**MAIN RESULTS AND THE ROLE OF CHANCE:**

Of the original 901 patients, 93 who were outliers for FSH or proliferative phase duration data were excluded from the analysis. Patients were classified according to quartiles of EMT on day of the LH surge, as follows: EMT < 8.1 mm, 8.1 mm ≤ EMT < 9.1 mm, 9.1 mm ≤ EMT < 10.6 mm and EMT ≥ 10.6 mm. Decreased EMT on day of the LH surge was associated with lower live birth (*P* = 0.0016) and higher chemical pregnancy (*P* = 0.0011) rates. Similarly, patients were classified according to quartiles of EMT on day of the SVBT, as follows: EMT < 9.1 mm, 9.1 mm ≤ EMT < 10.1 mm, 10.1 mm ≤ EMT < 12.1 mm and EMT ≥ 12.1 mm. A decreased EMT on the day of SVBT was associated with a lower live birth rate (*P* = 0.0095) but not chemical pregnancy rate (*P *=* *0.1640). Additionally, multivariate logistic regression analysis revealed a significant correlation between EMT on day of the LH surge and ongoing pregnancy; however, no correlation was observed between EMT on the day of SVBT and ongoing pregnancy (adjusted odds ratio 0.952; 95% CI, 0.850–1.066; *P *=* *0.3981). A decreased EMT on day of the LH surge was significantly associated with greater female age (*P *=* *0.0003) and a shortened follicular/proliferation phase (*P *<* *0.0001).

**LIMITATIONS, REASONS FOR CAUTION:**

The data used in this study were obtained from a single-centre cohort; therefore, multi-centre studies are required to ascertain the generalisability of these findings to other clinics with different protocols and/or patient demographics.

**WIDER IMPLICATIONS OF THE FINDINGS:**

This is the first report demonstrating a significant correlation between EMT on day of the LH surge and pregnancy outcomes after frozen blastocyst transfer in modified natural cycles. Our results suggest that EMT on day of the LH surge may be an effective predictor of the live birth rate.

**STUDY FUNDING/COMPETING INTEREST(S):**

This study was supported by resources from the Kato Ladies Clinic. The authors have no conflicts of interest to declare.

WHAT DOES THIS MEAN FOR PATIENTS?

The thickness of the innermost lining of the uterus (the endometrium) is normally increased by oestrogen in preparation for implantation of an embryo. This study evaluates whether endometrial thickness (EMT) on the day of LH surge (a rise in the hormone LH before ovulation) provides useful information on pregnancy outcomes in women who undergo frozen embryo transfer (ET). Currently, this information is unknown. This study also looks at the relationship between EMT (measured by ultrasound) on the day of ET and pregnancy outcomes, as recent studies have cast doubt on the usefulness of this measure in predicting pregnancy outcomes after IVF.

We observed that EMT on the day of the LH surge was better than that on the day of ET for predicting pregnancy outcomes. Our results suggest that the increase in EMT before ovulation is essential for improving implantation and live birth in women who undergo frozen ETs in natural cycles.

## Introduction

Endometrial thickness (EMT) measurement is routinely performed during IVF treatment because of its simple and non-invasive approach through transvaginal ultrasonography (Kasius *et al.*, 2014; Craciunas *et al.*, 2019). Previous studies demonstrated that increased EMT on the day of embryo transfer (ET) resulted in better pregnancy outcomes after fresh ETs (Vera *et al.*, 1995; Schwartz *et al.*, 1997; Kovacs *et al.*, 2003) and frozen ETs (Abdalla *et al.*, 1994; Schwartz *et al.*, 1997). However, recent studies reported that EMT on the day of ET is a poor predictor of IVF treatment outcomes (Polanski *et al.*, 2016; Griesinger *et al.*, 2018; Craciunas *et al.*, 2019); therefore, the predictive capacity of EMT on the day of ET is uncertain. In contrast, several studies showed that EMT on the day of the hCG trigger correlated with pregnancy outcomes after fresh ETs (McWilliams and Frattarelli, 2007; Richter *et al.*, 2007; Al-Ghamdi *et al.*, 2008; Traub *et al.*, 2009; Zhao *et al.*, 2014; Yuan *et al.*, 2016); thus, EMT on the day of the hCG trigger could be used as an indicator of endometrial receptivity and clinical pregnancy after fresh ET cycles. A similar association was also determined between pregnancy outcomes after frozen ETs and EMT on the day of progesterone initiation in HRT cycles (Noyes *et al.*, 2001; Zhang *et al.*, 2018). However, it remains unknown whether EMT on day of the LH surge is associated with pregnancy outcomes after frozen blastocyst transfer in natural cycles.

In ART, it has become increasingly common to culture and freeze all embryos at the expanded blastocyst stage (Shapiro *et al.*, 2014; Wei *et al.*, 2019). Compared with HRT cycles, frozen blastocyst transfers in natural cycles are preferable for women with regular menstrual cycles because it requires less medication and results in less financial burden on the patients (Agha-Hosseini *et al.*, 2018). Thus, in our clinic, the main strategy for frozen blastocyst transfers in patients with regular menstrual cycles revolves around the modified natural cycle. Before the administration of a GnRH agonist (GnRHa), which regulates the timing of oocyte maturation and ovulation in modified natural cycles, we evaluate the endogenous LH surge and follicular development, as well as the EMT, by transvaginal ultrasonography in women who undergo frozen blastocyst transfers. This study assessed the predictive capacity of the EMT on day of the LH surge in terms of the ongoing pregnancy rate after single vitrified-warmed blastocyst transfers (SVBTs). Furthermore, factors affecting the EMT on day of the LH surge were determined by evaluating the correlations between the EMT and the patient and cycle characteristics.

## Materials and methods

### Ethical approval

This retrospective cohort study was approved by the Institutional Review Board of Kato Ladies Clinic (approval number: 19–39). Written informed consent for the analysis of de-identified data was obtained from all patients.

### Patients

The records of 808 treatment cycles from 808 women, who underwent SVBTs in modified natural cycles at Kato Ladies Clinic between November 2018 and October 2019, were reviewed. Patients using autologous oocytes were included. Fifty-four patients with recurrent implantation failure (four or more unsuccessful ETs (Coughlan *et al.*, 2014)) were excluded. Fifty patients with the outliers for serum FSH level (<1.5 and >18 mIU/ml) and 43 patients with the outliers for proliferative phase duration (<8 and >18 days) were excluded.

### Embryo warming

Embryo warming was performed using Cryotop (Kitazato Corporation), as described previously (Kato *et al.*, 2014; Mori *et al.*, 2015). Briefly, the Cryotop was placed into a warming solution of 1.0 M sucrose at 37°C for 1 min. Subsequently, the blastocysts were removed from the warming solution and transferred to a diluent solution of 0.5 M sucrose at room temperature. After 3 min, they were transferred to the washing solution without sucrose. For final dilution, blastocysts were transferred to the washing solution for 1 min.

### Measurement of the EMT and ET

In the modified natural cycle SVBT protocol, the only pharmaceutical intervention involved the induction of final oocyte maturation with GnRHa. Monitoring included transvaginal ultrasonography and blood hormone (estradiol and progesterone) testing performed on Days 5–21, according to the patient’s cycle length. Ovulation was triggered using buserelin (Suprecur; Mochida Pharmaceutical Co., Ltd., Tokyo, Japan or Buserecur; Fuji Pharma Co., Ltd.), a nasal spray containing GnRHa, after confirming initiation of the LH surge. The EMT was measured through transvaginal ultrasonography on the day of LH surge (Nishihara *et al.*, 2020). Blastocysts were selected according to their developmental speed and morphological grade (Kato *et al.*, 2014), and SVBTs were performed (Kato *et al.*, 2018). The EMT was measured by transvaginal ultrasonography immediately before the SVBT procedure. Dydrogesterone (30 mg/day) was administered orally during the early luteal phase after SVBT. In addition, in cases with insufficient luteal function (progesterone level on the day of SVBT <11 ng/ml), progesterone was administered intravaginally (Lutinus, Ferring Pharmaceuticals, Saint Prex, Switzerland) until the ninth week of pregnancy. Implantation was defined by serum hCG level (>20 IU/ml), in accordance with a previous study (Ueno *et al.*, 2014). The clinical and ongoing pregnancy rates were defined according to the ultrasonographic observation of a gestational sac 3 weeks after SVBTs and the observation of a foetal heartbeat 5 weeks after SVBTs, respectively (Ezoe *et al.*, 2020).

### Statistical analyses

Statistical analyses were performed using JMP software (SAS, Cary, NC, USA). Proportional data were analysed using the Cochran–Armitage test for trends and the chi-square test. Continuous parameters were compared using ANOVA, and statistical significance was determined using Tukey’s test for *post-hoc* analysis. The Spearman rank-order correlation test was used to measure the degree of association between two continuous variables. Univariate logistic regression analysis was used to identify the covariates that were potentially associated with live birth. The likelihood ratio test for the significance of the coefficient was performed, and the variables with *P *<* *0.05 were used as confounders. Similarly, multivariate logistic regression analysis for the live birth was used to adjust the bias (using the confounders) and verify the statistical significance (using Wald statistic). Odds ratios (ORs) and adjusted ORs (AORs) are reported with 95% CIs for each group. The goodness of fit of the multivariate logistic regression analysis was evaluated using Pearson Chi-square statistic. Receiver operating characteristic (ROC) curve analysis was performed, and AUC, reflecting the discrimination power of the model, was calculated (Zhang, 2016). A value of *P *<* *0.05 was considered statistically significant.

## Results

### Patient characteristics and clinical outcomes

Table I shows the patients’ demographic and cycle characteristics. During the study period, 808 patients underwent 808 SVBT cycles. The mean ages of the women and men were 38.2 ± 0.1 and 40.3 ± 0.2 years, respectively. EMT values on the days of LH surge and SVBT were 9.4 ± 0.1 mm (5.0–17.4 mm) and 10.4 ± 0.1 mm (6.0–20.0 mm), respectively. Among the 808 blastocysts transferred, 280 (34.7%) were produced by conventional IVF (cIVF), and 528 (65.3%) were produced by ICSI. The rates of clinical pregnancy, ongoing pregnancy and live birth after SVBTs were 47.4% (383/808), 41.0% (331/808) and 34.9% (282/808), respectively. The rates of chemical pregnancy and miscarriage were 11.5% (50/433) and 26.4% (101/383), respectively.

### Correlations between the EMT and pregnancy outcomes

#### EMT on the day of LH surge

Patients were stratified into four groups, according to quartiles of the EMT, on the day of LH surge as follows: first quartile, EMT < 8.1 mm; second quartile, 8.1 mm ≤ EMT < 9.1 mm; third quartile, 9.1 mm ≤ EMT < 10.6 mm; and fourth quartile, 10.6 mm ≤ EMT (Table II). Clinical pregnancy, ongoing pregnancy and live birth rates after SVBT significantly increased with the EMT increase from the first to the fourth quartile group. The Cochran–Armitage test confirmed that clinical pregnancy, ongoing pregnancy and live birth rates decreased with a decrease in the EMT on the day of LH surge (*P *=* *0.0069, *P *=* *0.0041, and *P *=* *0.0016, respectively). A higher chemical pregnancy rate was associated with a thinner endometrium on the day of LH surge (*P *=* *0.00111). Furthermore, the EMT on the day of LH surge negatively correlated with the female age.

**Table I:** Patient characteristics and pregnancy outcomes.

No. of patients included, n	901
No. of patients excluded, n	93
No. of patients analysed, n	808
Female age, years, mean ± SEM (range)	38.2 ± 0.1 (24–47)
Male age, years, mean ± SEM (range)	40.3 ± 0.2 (26–65)
No. of previous ET cycles, mean ± SEM (range)	1.0 ± 0.0 (0–3)
Infertility cause, n (%)	
Ovulation factor	4 (0.5)
Oviduct factor	51 (6.3)
Endometrial factor	95 (11.8)
Male factor	129 (16.0)
Combination	43 (5.3)
Unexplained	486 (60.1)
Basal serum hormonal level	
Estradiol (pg/ml), mean ± SEM (range)	35.7 ± 0.7 (4–83)
Progesterone (ng/ml), mean ± SEM (range)	0.1 ± 0.0 (0.0–0.2)
FSH (mIU/ml), mean ± SEM (range)	9.4 ± 0.1 (1.6–19.5)
LH (mIU/ml), mean ± SEM (range)	3.7 ± 0.1 (0.1–6.5)
No. of ET cycles	808
Length of proliferation (follicular) phase, from menstruation initiation to LH surge	12.9 ± 0.1 (8–18)
Endometrial thickness on the day of LH surge (mm)	9.4 ± 0.1 (5.0–17.4)
Estradiol on the day of LH surge (pg/ml)	257.9 ± 2.7 (169–492)
Progesterone on the day of LH surge (ng/ml)	0.3 ± 0.0 (0.0–1.5)
Endometrial thickness on the day of SVBT (mm)	10.4 ± 0.1 (6.0–20.0)
Estradiol on the day of SVBT (pg/ml)	164.7 ± 2.1 (48–414)
Progesterone on the day of SVBT (ng/ml)	17.3 ± 0.2 (9.1–35.6)
Insemination	
cIVF	280 (34.7)
ICSI	528 (65.3)
Culture time (time required for the blastocyst expansion) (h)	123.9 ± 0.4 (98–169)
Morphological grade of the transferred blastocysts, n (%)	
ICM	
Grade A, n (%)	306 (37.9)
Grade B, n (%)	296 (36.6)
Grade C, n (%)	206 (25.5)
TE	
Grade A, n (%)	291 (36.0)
rade B, n (%)	219 (27.1)
Grade C, n (%)	298 (36.9)
Clinical pregnancy, n (%)	383 (47.4)
Ongoing pregnancy, n (%)	331 (41.0)
Live birth, n (%)	282 (34.9)
Chemical pregnancy, n (%)	50 (11.5)
Miscarriage, n (%)	101 (26.4)

cIVF, conventional IVF; SVBT, single vitrified-warmed blastocyst transfer; ICM, inner cell mass; TE, trophectoderm; ET, embryo transfer.

#### EMT on the day of SVBT

Patients were stratified into four groups, according to quartiles of the EMT, on the day of SVBT: first quartile, EMT < 9.1 mm; second quartile, 9.1 mm ≤ EMT < 10.1 mm; third quartile, 10.1 mm ≤ EMT < 12.1 mm; and fourth quartile, 12.1 mm ≤ EMT (Table III). The clinical pregnancy, ongoing pregnancy and live birth rates after SVBT significantly increased with the EMT increase from the first to the fourth quartile group. The Cochran–Armitage test confirmed that both clinical and ongoing pregnancy rates decreased with a decrease in the EMT on the day of SVBT (*P *=* *0.0265, *P *=* *0.0075 and *P *=* *0.0095, respectively). However, the chemical pregnancy and miscarriage rates did not correlate with the EMT on the day of SVBT (*P *=* *0.1640 and *P *=* *0.4652, respectively). Decreased EMT on the day of SVBT was similarly associated with the female age.

**Table II:** Pregnancy outcomes after SVBT, stratified by endometrial thickness on the day of the LH surge.

Quartile group	No. of cycles	Female age	Implantation (%)	Clinical pregnancy (%)	Ongoing pregnancy (%)	Live birth (%)	Chemical pregnancy (%)	Miscarriage (%)
First: EMT <8.1 mm	237	39.0 ± 0.2^a^	113 (47.7)	93 (39.2)^a^	78 (32.9)^a^	62 (26.2)^a^	20 (17.7)^a^	31 (33.3)
Second: 8.1 mm ≤ EMT <9.1 mm	179	38.0 ± 0.3^b^	101 (56.4)	83 (46.4)^a, b^	70 (39.1)^a, b^	61 (34.1)^a, b^	18 (17.8)^a^	22 (26.5)
Third: 9.1 mm ≤ EMT <10.6 mm	205	38.1 ± 0.3^b^	109 (53.2)	103 (50.2)^b^	90 (43.9)^b, c^	77 (37.6)^b^	6 (5.5)^b^	26 (25.2)
Fourth: 10.6 mm ≤ EMT	187	37.6 ± 0.3^b^	110 (55.6)	104 (54.9)^b^	93 (49.7)^c^	82 (43.9)^b^	6 (5.5)^b^	22 (21.2)
*P*-value	–	0.0009	0.1125	0.0069	0.0041	0.0016	0.0011	0.2784

EMT, endometrial thickness; SVBT, single vitrified-warmed blastocyst transfer.

^a–c^Different superscript letters indicate a significant difference at *P* < 0.05 (chi-square test).

**Table IV:** **Multivariate logistic regression analysis for live birth.** ^*^

	Adjusted odds ratio	95% CI	*P*-value	AUC
Female age	0.823	0.778–0.870	<0.0001	0.795
Male age	0.989	0.952–1.026	0.5545	
Culture time	0.958	0.939–0.979	<0.0001	
Gardner's Criteria				
ICM				
Grade A	Reference	–	–	
Grade B	1.001	0.675–1.482	0.9971	
Grade C	1.080	0.611–1.909	0.7923	
TE				
Grade A	Reference	–	–	
Grade B	0.511	0.338–0.773	0.0015	
Grade C	0.319	0.190–0.536	<0.0001	
EMT on the day of LH surge^**^	1.188	1.057–1.338	0.0037	
EMT on the day of SVBT^**^	0.952	0.850–1.066	0.3981	

* Goodness of fit test: χ^2^ = 199.24, df = 9, *P*-value <0.0001.

** The EMT on the day of LH surge and SVBT were used as the continuous variables in this analysis.

SVBT, single vitrified-warmed blastocyst transfer; EMT, endometrial thickness; ICM, inner cell mass; TE, trophectoderm.

#### Multivariate logistic regression analysis for pregnancy outcomes

The univariate logistic analysis revealed significant associations between the ongoing pregnancy rate and female and male ages, embryo culture time, blastocyst morphology and the EMT on the days of LH surge and ET (Supplementary Table SI). The multivariate logistic regression analysis was performed to adjust for potential statistical co-founding biases (Table IV). Decreased EMT on the day of LH surge was significantly associated with a lower ongoing pregnancy rate (AOR, 1.188; 95% CI, 1.057–1.338; *P* = 0.0037), even after adjustment for confounders; however, no statistical association was observed between the ongoing pregnancy rate and the EMT on the day of SVBT after adjustment for confounders (AOR, 0.952; 95% CI, 0.850–1.066; *P* = 0.3981).

**Table III:** Pregnancy outcomes after SVBT stratified by EMT on the day of SVBT.

Quartile group	No. of cycles	Female age	Implantation (%)	Clinical pregnancy (%)	Ongoing pregnancy (%)	Live birth (%)	Chemical pregnancy (%)	Miscarriage (%)
First: EMT <9.1 mm	211	39.4 ± 0.2^a^	101 (47.9)	88 (41.7)^a^	74 (35.1)^a^	62 (29.4)^a^	13 (12.9)^a^	26 (29.6)
Second: 9.1 mm ≤ EMT <10.1 mm	300	38.2 ± 0.2^b^	158 (52.7)	135 (45.0)^a, b^	117 (39.0)^a^	96 (32.0)^a, b^	23 (14.6)^a^	39 (28.9)
Third: 10.1 mm ≤ EMT <12.1 mm	207	37.7 ± 0.3^b, c^	120 (58.0)	108 (52.2)^b, c^	90 (43.5)^a, b^	82 (39.6)^b, c^	12 (10.0)^b^	26 (24.1)
Fourth: 12.1 mm ≤ EMT	90	36.8 ± 0.4^c^	54 (60.0)	52 (57.8)^c^	50 (55.6)^b^	42 (46.7)^c^	2 (3.7)^b^	10 (19.2)
*P*-value	–	<0.0001	0.1133	0.0265	0.0075	0.0095	0.1640	0.4652

^a–c^ Different superscript letters indicate a significant difference at *P* < 0.05 (chi-square test).

SVBT, single vitrified-warmed blastocyst transfer; EMT, endometrial thickness.

Patients were stratified by the median EMT value on the day of LH surge (9.1 mm) and further stratified, according to the quartile of EMT, on the day of SVBT (Supplementary Table SII). When the EMT on the day of LH surge was <9.1 mm, the clinical pregnancy, ongoing pregnancy and live birth rates were comparable among the groups, regardless of the EMT on the day of SVBT.

### Factors affecting the EMT on the day of LH surge

Associations between the EMT on the day of LH surge and patient/cycle characteristics were analysed (Supplementary Table SIII). The number of previous ET cycles, basal estradiol, progesterone, FSH level and LH and serum levels of estradiol and progesterone on the day of LH surge were not associated with the EMT on the day of LH surge. In contrast, the female age and the length of the follicular phase significantly correlated with the EMT on the day of LH surge. Furthermore, the female age was negatively associated with the follicular phase length. The infertility cause was not associated with the EMT on the day of LH surge (Supplementary Fig. S1).

## Discussion

The predictive value of EMT on day of the LH surge in terms of pregnancy outcomes after frozen blastocyst transfers has not been determined. However, the EMT values on the day of the hCG trigger in fresh ET cycles and the day of progesterone initiation in HRT cycles have been reported as predictors of pregnancy outcomes (Noyes *et al.*, 2001; McWilliams and Frattarelli, 2007; Richter *et al.*, 2007; Al-Ghamdi *et al.*, 2008; Traub *et al.*, 2009; Harlow and Paramsothy, 2011; Zhao *et al.*, 2014; Yuan *et al.*, 2016). Our study provides the first evidence that decreased EMT on the day of LH surge can result in a reduced live birth rate after SVBT. Furthermore, the EMT on the day of LH surge was significantly associated with female age and the length of the proliferation (follicular) phase.

In the human endometrium, steroid hormones secreted from the ovaries regulate cell division, differentiation and degeneration. The menstrual cycle is classified into a menstrual phase, a proliferation phase (before ovulation), and a secretory phase (after ovulation). In the menstrual and proliferation phases, the functional layer is repaired under the influence of oestrogen, and the EMT increases in preparation for embryo implantation (Groothuis *et al.*, 2007; Cha *et al.*, 2012). In the secretory phase, progesterone inhibits oestrogen-induced proliferation and allows stromal cells to begin decidualization; thus, the functional layer of the endometrium thickens in preparation for embryo implantation (Marquardt *et al.*, 2019), and the increase in EMT on the day of LH surge is strongly affected by oestrogen secreted during the proliferation phase, reflecting the degree of proliferation (quantity of endometrium). In contrast, the increase in EMT on the day of SVBT is affected by both oestrogen and progesterone secreted during the secretory phase and reflects the degree of differentiation (endometrium quality). This implies that the biological significance of an increase in EMT is different between the proliferation and secretory phases. Univariate analysis showed that both EMTs were significantly associated with clinical pregnancy, ongoing pregnancy and live birth rates and negatively correlated with the female age. In contrast, multivariate logistic regression analysis revealed that the EMT on the day of LH surge (rather than the day of SVBT) was significantly associated with the live birth rate after SVBT and correlated with the chemical pregnancy rate. These results indicate that, compared with EMT on the day of SVBT, the EMT on the day of LH surge is a better predictor of pregnancy outcomes after frozen blastocyst transfers in modified natural cycles. Similarly, our data showed that when the EMT on the day of LH surge was <9.1 mm, pregnancy outcomes were significantly impaired even when the EMT on the day of SVBT was adequately thick (>10 mm). These results suggest that the oestrogen-induced EMT increase during the proliferation phase is essential for the improvement of uterine receptivity and the success of implantation, clinical pregnancy and live birth.

We further analysed the factors potentially affecting the EMT on the day of LH surge. Our results demonstrated that decreased EMT on the day of LH surge was negatively correlated with the female age and positively associated with the length of the proliferation phase. Furthermore, female age was significantly associated with a shortened proliferation phase, which is consistent with findings in previous studies (Shapley *et al.*, 2004; Van Voorhis *et al.*, 2008; Harlow and Paramsothy, 2011; Mumford *et al.*, 2012). A prior study similarly showed that short ovulatory cycles were associated with an earlier rise in FSH and oestrogen, which is often observed in older patients. This same study reported that a shortened proliferation phase leads to insufficient accumulative exposure to estradiol, LH and FSH (Mumford *et al.*, 2012). During the proliferation phase, endometrial cell proliferation is promoted by oestrogen and peaks at around 8 − 10 days from the onset of menstruation (Ferenczy *et al.*, 1979; Couse and Korach, 1999; Groothuis *et al.*, 2007). In addition, exposure to oestrogen for at least 5 days is required for endometrial thickening and implantation (Navot and Bergh, 1991; Michalas *et al.*, 1996). Furthermore, exposure to oestrogen during the proliferation phase reportedly increases the sensitivity to progesterone and thickening of the functional layer in the implantation period (Groothuis *et al.*, 2007). Therefore, on the day of LH surge, patients with a thin endometrium caused by a shortened proliferation phase may show not only inadequate endometrial proliferation but also decreased progesterone sensitivity.

The main strength of this study was that the data were obtained from a single-centre cohort of patients who underwent single blastocyst transfers in modified natural cycles; however, several limitations should also be mentioned. The study is retrospective in nature, and further multi-centre studies are required to ascertain the generalisability of these findings to other clinics with different protocols and/or patient demographics.

In conclusion, we demonstrated that the EMT on the day of LH surge was significantly associated with clinical pregnancy, ongoing pregnancy and live birth rates after frozen blastocyst transfers. Similarly, our results demonstrated significant associations between the EMT on the day of LH surge and a shortened proliferation phase induced by female ageing. Therefore, our results suggest that EMT on the day of LH surge could be used as an effective predictor of pregnancy outcomes. Previous studies reported that there is a significant shortening of the menstrual cycle with age due to a decrease in the length of the follicular phase, and this irregularity can be improved using hormonal contraception (Baird *et al.*, 2005; ESHRE Capri Workshop Group, 2008, 2009). Therefore, when a thin endometrium is observed during the proliferation phase, the ET should be cancelled. The endogenous FSH and LH suppression by exogenous ovarian steroid hormone (pill) administration followed by the ET in the next cycle, or re-scheduling of the HRT cycle, could improve pregnancy outcomes.

## Supplementary data

Supplementary data are available at *Human Reproduction Open* online.

## Data availability

The data underlying this article cannot be shared publicly due to the privacy concerns of individuals that participated in the study. The data will be shared on reasonable request to the corresponding author subject to local regulatory approvals.

## Authors’ roles

S.O. contributed to the data collection, interpretation and writing. K.E. contributed to the study design, analysis, interpretation and writing. S.N. contributed to the data collection, interpretation and writing. J.F. contributed to interpretation and writing. T.K. contributed to manuscript revision. K.K. contributed to the study design, interpretation and writing. All authors read and approved the final manuscript.

## Funding

This research did not receive any specific grant from funding agencies in the public, commercial or not-for-profit sectors.

## Conflict of interest

The authors have no conflicts of interest to declare.

## Supplementary Material

hoaa060_Supplementary_Data
