# Supplementary material for: Endometrial thickness on the day of the LH surge: an effective predictor of pregnancy outcomes after modified natural cycle-frozen blastocyst transfer
Source: Hum Reprod Open. 2020 Dec 17;2020(4):hoaa060. doi: 10.1093/hropen/hoaa060 (PMC7821991; doi:10.1093/hropen/hoaa060)
Supplement: hoaa060_Supplementary_Data [file hoaa060_supplementary_data.zip › Supplementary Tables final.docx]

**Supplementary Table SI** Univariate logistic regression analysis for live birth.

|  | Odds ratio | 95% CI | P value | AUC |
| --- | --- | --- | --- | --- |
| Female age | 0.809 | 0.774–0.843 | < 0.0001 | 0.723 |
| Male age | 0.915 | 0.888–0.941 | < 0.0001 | 0.638 |
| No. of previous ET | 0.927 | 0.797–1.076 | 0.3225 | 0.515 |
| Insemination |  |  |  | 0.500 |
| cIVF | Reference | – |  |  |
| ICSI | 0.993 | 0.733–1.346 | 0.9657 |  |
| Culture time | 0.936 | 0.918–0.951 | < 0.0001 | 0.652 |
| Gardner's Criteria |  |  |  |  |
| ICM Grade A | Reference | – | – | 0.618 |
| Grade B | 0.605 | 0.434–0.840 | 0.0027 |  |
| Grade C | 0.303 | 0.200–0.452 | < 0.0001 |  |
| TE Grade A | Reference | – | – | 0.672 |
| Grade B | 0.476 | 0.331–0.682 | < 0.0001 |  |
| Grade C | 0.207 | 0.141–0.298 | < 0.0001 |  |
| EMT on the day of LH surge* | 1.167 | 1.078–1.265 | 0.0002 | 0.581 |
| EMT on the day of SVBT* | 1.130 | 1.047–1.220 | 0.0016 | 0.568 |

ET, embryo transfer; cIVF, conventional IVF; ICM, inner cell mass; TE, trophectoderm; EMT, endometrial thickness; SVBT, single vitrified-warmed blastocyst transfer.

* Endometrial thickness on the day of LH surge and SVBT was used as the continuous variable in this analysis.

**Supplementary Table SII** Pregnancy outcomes after single vitrified-warmed blastocyst transfer, stratified by the endometrial thicknesses on the day of LH surge and SVBT.

| Endometrial thickness  on the day of LH surge | Endometrial thickness  on the day of SVBT | No. of  cycles | Implantation  (%) | Clinical  pregnancy  (%) | Ongoing  pregnancy  (%) | Live birth  (%) |
| --- | --- | --- | --- | --- | --- | --- |
| < 9.1 mm | - | 416 | 214 (51.4) | 176 (42.3) | 148 (35.6) | 123 (29.6) |
| < 9.1 mm | <9.1 mm | 176 | 85 (48.3) | 73 (41.5) | 62 (35.2) | 53 (30.1) |
| < 9.1 mm | 9.1 mm≤, <10.1 mm | 163 | 86 (52.8) | 68 (41.7) | 58 (35.6) | 44 (27.0) |
| < 9.1 mm | 10.1 mm≤, <12.1 mm | 65 | 36 (55.4) | 29 (44.6) | 24 (36.9) | 22 (33.9) |
| < 9.1 mm | 12.1 mm≤ | 12 | 7 (58.3) | 6 (50.0) | 4 (33.3) | 4 (33.3) |
| 9.1 mm ≤ | - | 392 | 219 (55.9) | 207 (52.8) | 183(46.7) | 159 (40.6) |
| 9.1 mm ≤ | <10.1 mm | 172 | 88 (51.1) | 82 (47.7) | 71 (41.2)^a^ | 61 (35.5)^a^ |
| 9.1 mm ≤ | 10.1 mm≤, <12.1 mm | 142 | 84 (59.1) | 79 (55.6) | 66 (46.5)^a,b^ | 60 (42.3)^a,b^ |
| 9.1 mm ≤ | 12.1 mm≤ | 78 | 47 (60.2) | 46 (59.0) | 46 (59.0)^b^ | 38 (48.7)^b^ |

^a–b^ Different superscript letters indicate a significant difference at P < 0.05 (chi-square test).

**Supplementary Table SIII** Correlation of the endometrial thickness on the day of LH surge with the cycle characteristics.

| Parameter I | Parameter II | Spearman's rank  correlation coefficient | *P* value |
| --- | --- | --- | --- |
| Endometrial thickness on the day of LH surge* | Female age | -0.1276 | 0.0003 |
|  | No. of previous embryo transfer cycles | -0.0635 | 0.0711 |
|  | Basal estradiol level | -0.013 | 0.7117 |
|  | Basal progesterone level | 0.005 | 0.8875 |
|  | Basal FSH level | -0.0226 | 0.5206 |
|  | Basal LH level | 0.0351 | 0.3186 |
|  | Estradiol level on the day of LH surge | -0.0248 | 0.4825 |
|  | Progesterone level on the day of LH surge | 0.0513 | 0.146 |
|  | Length of the proliferation (follicular) phase | 0.1378 | < 0.0001 |
| Female age | Length of the proliferation (follicular) phase | -0.1146 | 0.0011 |

The endometrial thickness on the day of LH surge and SVBT was used as the continuous variable in this analysis.
