# Supplementary figures and images for: Endometrial thickness on the day of the LH surge: an effective predictor of pregnancy outcomes after modified natural cycle-frozen blastocyst transfer
Source: Hum Reprod Open. 2020 Dec 17;2020(4):hoaa060. doi: 10.1093/hropen/hoaa060 (PMC7821991; doi:10.1093/hropen/hoaa060)

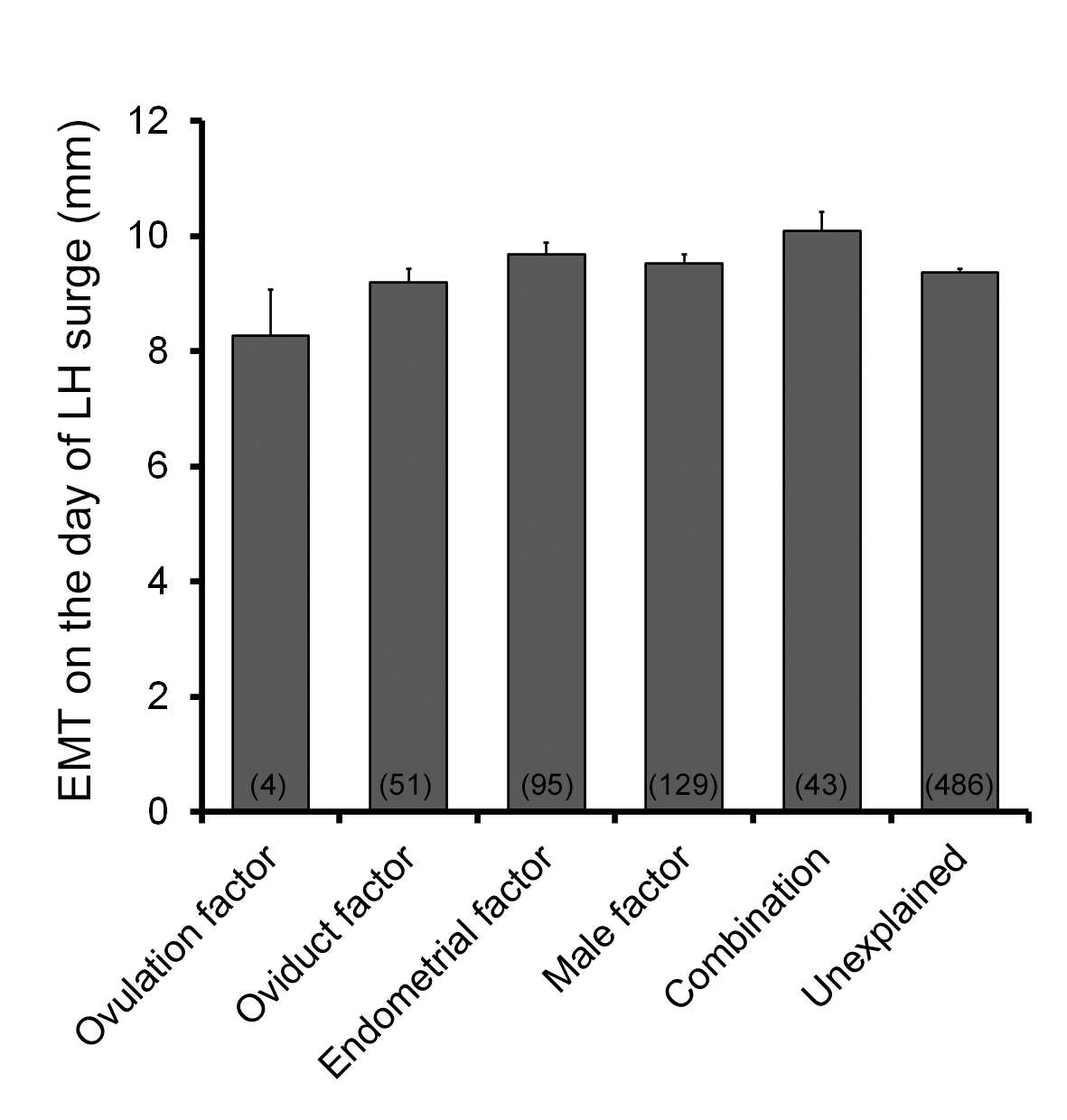

Supplement: hoaa060_Supplementary_Data [file hoaa060_supplementary_data.zip › Supplementary Figure S1 final.tif]
